# Anti-*Acanthamoeba* synergistic effect of chlorhexidine and *Garcinia mangostana* extract or α-mangostin against *Acanthamoeba triangularis* trophozoite and cyst forms

**DOI:** 10.1038/s41598-021-87381-x

**Published:** 2021-04-13

**Authors:** Suthinee Sangkanu, Watcharapong Mitsuwan, Wilawan Mahabusarakam, Tajudeen O. Jimoh, Polrat Wilairatana, Ana Paula Girol, Ajoy K. Verma, Maria de Lourdes Pereira, Mohammed Rahmatullah, Christophe Wiart, Abolghasem Siyadatpanah, Roghayeh Norouzi, Polydor Ngoy Mutombo, Veeranoot Nissapatorn

**Affiliations:** 1 School of Allied Health Sciences, Southeast Asia Water Team (SEA Water Team), World Union for Herbal Drug Discovery (WUHeDD), and Research Excellence Center for Innovation and Health Products (RECIHP), Walailak University, Nakhon Si Thammarat, Thailand; 2 Akkhraratchakumari Veterinary College and Research Center of Excellence in Innovation of Essential Oil, Walailak University, Nakhon Si Thammarat, Thailand; 3 Division of Physical Science, Faculty of Science, Prince of Songkla University, Songkhla, Thailand; 4 Department of Pharmacognosy and Pharmaceutical Botany, Faculty of Pharmaceutical Sciences, Chulalongkorn University, Bangkok, Thailand; 5 Department of Biochemistry, Habib Medical School, Islamic University in Uganda, Kampala, Uganda; 6 Department of Clinical Tropical Medicine, Faculty of Tropical Medicine, Mahidol University, Bangkok, Thailand; 7 Department of Biology, Faculty of Sciences, São Paulo State University, São Paulo, Brazil; 8 Department of Microbiology, National Institute of Tuberculosis & Respiratory Diseases (NITRD), New Delhi, India; 9 Department of Medical Sciences, CICECO-Aveiro Institute of Materials &, University of Aveiro, Aveiro, Portugal; 10 Department of Biotechnology & Genetic Engineering, University of Development Alternative Lalmatia, Dhaka, Bangladesh; 11 School of Pharmacy, University of Nottingham Malaysia Campus, Selangor, Malaysia; 12 Ferdows School of Paramedical and Health, Birjand University of Medical Sciences, Birjand, Iran; 13 Department of Pathobiology, Faculty of Veterinary Medicine, University of Tabriz, Tabriz, Iran; 14 School of Public Health and Community Medicine, UNSW Medicine, UNSW, Sydney, NSW Australia; 15 Centre for Biomedical Research, Burnet Institute, Melbourne, VIC Australia

**Keywords:** Drug discovery, Plant sciences, Diseases, Health care, Pathogenesis

## Abstract

*Acanthamoeba* spp. can cause amoebic keratitis (AK). Chlorhexidine is effective for AK treatment as monotherapy, but with a relative failure on drug bioavailability in the deep corneal stroma. The combination of chlorhexidine and propamidine isethionate is recommended in the current AK treatment. However, the effectiveness of treatment depends on the parasite and virulence strains. This study aims to determine the potential of *Garcinia mangostana* pericarp extract and α-mangostin against *Acanthamoeba triangularis*, as well as the combination with chlorhexidine in the treatment of *Acanthamoeba* infection. The minimal inhibitory concentrations (MICs) of the extract and α-mangostin were assessed in trophozoites with 0.25 and 0.5 mg/mL, for cysts with 4 and 1 mg/mL, respectively. The MIC of the extract and α-mangostin inhibited the growth of *A. triangularis* trophozoites and cysts for up to 72 h. The extract and α-mangostin combined with chlorhexidine demonstrated good synergism, resulting in a reduction of 1/4–1/16 of the MIC. The SEM results showed that *Acanthamoeba* cells treated with a single drug and its combination caused damage to the cell membrane and irregular cell shapes. A good combination displayed by the extract or α-mangostin and chlorhexidine, described for the first time. Therefore, this approach is promising as an alternative method for the management of *Acanthamoeba* infection in the future.

## Introduction

*Acanthamoeba* is an opportunistic amoeba distributed in diverse natural habitats^1^. This organism have two main forms: the trophozoite, an invasive stage; and cyst, a highly resistant stage in a very harsh conditions^2^. Based on the size, shape and features of cysts, *Acanthamoeba* spp. have been divided into three groups (I, II, III). However, *Acanthamoeba* spp. has been classified into 22 different genotypes (T1–T22 Genotype) based on molecular technique, which used 18S rRNA gene sequencing^3–5^. Among them, T4 genotyge is the most isolated in clinical and environmental samples, followed by genotypes T3 and T5. In addition, the T4 genotype is the most virulent because it has a significant potential for binding to host cells than other genotypes^6^.

*Acanthamoeba* spp. are the causative agents of amoebic keratitis (AK) and granulomatous amoebic encephalitis (GAE). AK can cause permanent loss of vision^7^. The rate of infectious keratitis is becoming alarming in recent times, a problem that may be related to a sudden increase in the population of contact lens wearers^2^ Similarly, *Acanthamoeba* encysts penetrated deeply into the corneal stroma^8^ as such, the cyst wall becomes impervious to existing drugs, and this becomes a drawback for further studies in the areas of drug formulations and designed for this organism.

*Garcinia mangostana* Linn. is generally known as mangosteen and belongs to the family *Clusiaceae*. It is a tropical tree, widely distributed in Southeast Asia^9^. The pericarps of this fruit are commonly used in traditional medicine to treat several diseases which are non-toxic and safe to use^10,11^. Major compound in the mangosteen pericarps is xanthone group, particularly α-mangostin, which exhibited antibacterial activity^12^, antifungal activity^13^, antioxidant activity^14^ anti-cancer activity^15–17^, anti-inflammatory activity^18,19^, antiparasitic activity^20–22^.

To the best of our knowledge, we discover that there is no single report on the anti-*Acanthamoeba* activity of *G. mangostana* extract to date. Hence, our study sought to investigate the effective concentration of the *G. mangostana* extract and α-mangostin on the growth inhibition of *Acanthamoeba* spp. and to demonstrate its synergistic effects combined with chlorhexidine on anti-*Acanthamoeba* activity.

## Results

### Genotypic and species identification of *Acanthamoeba* isolate WU19001

**Figure 1:**
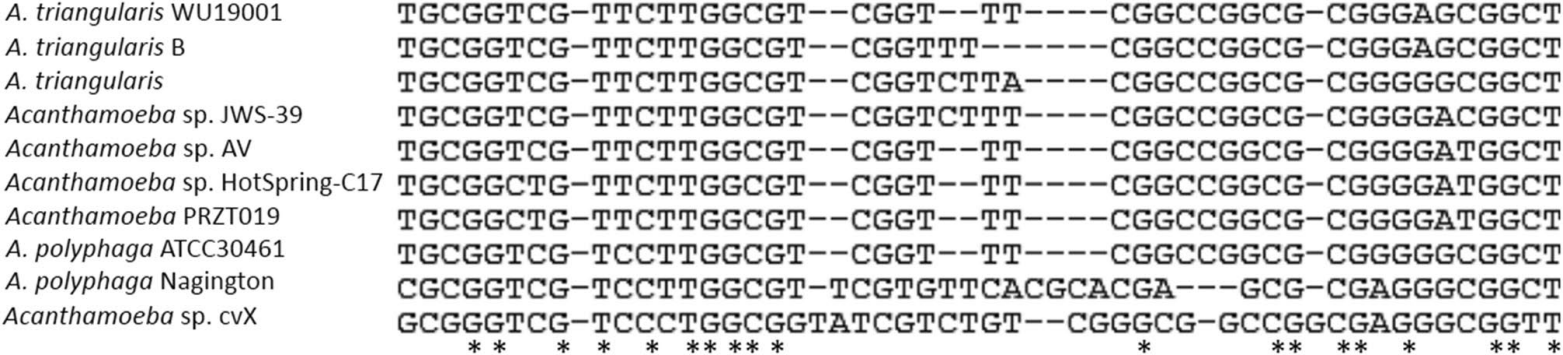
Primary sequence alignment of the DF3 region using CLUSTAL W. The region shown a subset of the total DF3 region which demonstrates the highest variation. Asterisks denote similarities and dashes denote gaps within the nucleotide sequences.

**Figure 2:**
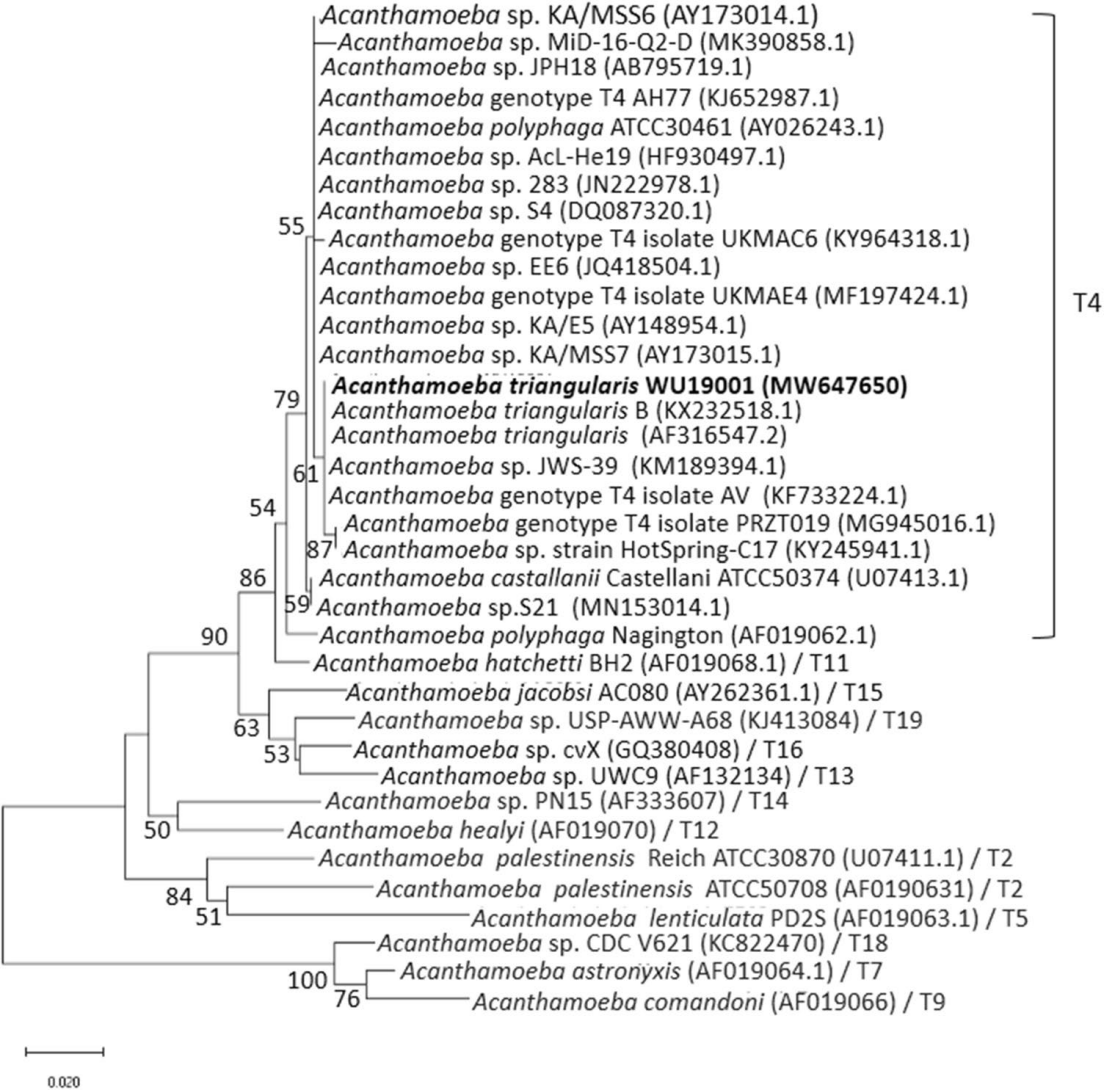
A phylogenetic tree was constructed using the neighbour-joining method with the Kimura two-parameter algorithm with bootstrapping values for 1000 replicates. The tree was reconstructed by considering 35 *Acanthamoeba* spp. isolates with the reference strains from NCBI.

The partial nucleotide sequences of DF3 region of *Acanthamoeba* sp. WU19001 from our previous study aligned using CLUSTAL W and revealed the highest variation, as shown in Fig. 1. The 18S rDNA sequences were subjected to phylogenetic analysis and species identification. It showed very similar patterns to *Acanthamoeba triangularis* KX232518.1 (99.74% similarity). The sequence homology search for the 35 *Acanthamoeba* spp. in the National Center for Biotechnology Information (NCBI) database showed WU19001 formed *Acanthamoeba* genotype T4 cluster (Fig. 2). The nucleotide sequence of WU19001 has been deposited at Genbank under the accession number MW647650.

### Minimum inhibitory concentration (MIC)

*G. mangostana* extract and α-mangostin were determined for their anti-*Acanthamoeba* potential by MIC using the microtiter dilution broth method. As shown in Table 1, the MICs of *G. mangostana* extract against *A. triangularis* trophozoites and cysts were 0.25 and 4 mg/mL. The pure compound, α-mangostin, exhibited MIC values at 0.5 and 1 mg/mL for trophozoites and cysts. The MIC values of chlorhexidine against trophozoites and cysts were 0.008 and 0.064 mg/mL, respectively.

**Table 1:** Minimal inhibitory concentration (MIC) of *G. mangostana* extract, α-mangostin and chlorhexidine against *A. triangularis* trophozoites and cysts.

Antimicrobial agents	MIC (mg/mL)	
	Trophozoites	Cysts
*G. mangostana* extract	0.25	4
α-mangostin	0.5	1
Chlorhexidine	0.008	0.064

### Growth assay

**Figure 3:**
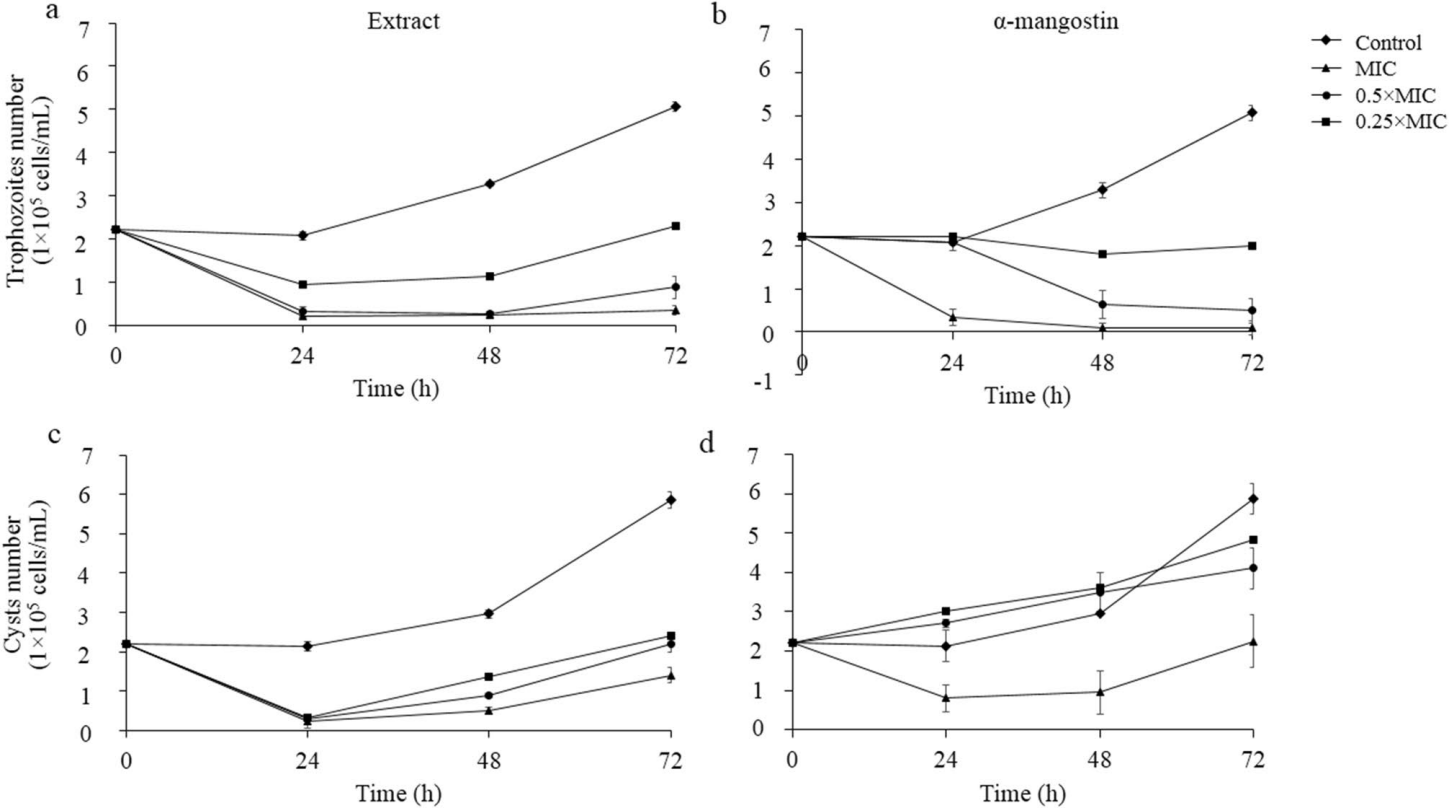
*G. mangostana* extract and α-mangostin inhibit the growth of *A. triangularis* trophozoites and cysts in vitro. To determine the effect of *G. mangostana* extract and α-mangostin on trophozoites (**a**,**b**) and on cysts (**c**,**d**), assays were performed by inoculating 2 × 10^5^ cells/mL in PYG medium in the presence of *G. mangostana* extract or α-mangostin at MIC, 0.5 × MIC and 0.25 × MIC final concentrations. The inhibitory activity was carried out using trypan blue exclusion assay up to 72 h; 1% DMSO was used as a negative control.

Susceptibilities of *A. triangularis* to *G. mangostana* extract and α-mangostin were determined by growth assay. *A. triangularis* was found to be significantly (*p* < 0.05) susceptible to the extract and α-mangostin at all concentrations when compared to control. The extract and α-mangostin showed higher susceptibility against *Acanthamoeba* trophozoites than cysts. (Fig. 3). After 72 h incubation, MIC values of the extract and α-mangostin could decrease the viability to 0.15 × 10^5^ and 0.1 × 10^5^ cells/mL of trophozoites as compared to the control (6.0 × 10^5^) (Fig. 3a,b).

After 24 h of incubation, the number of *Acanthamoeba* cysts decreased to 2.3 × 10^5^ and 0.8 × 10^5^ cells/mL when treated with MIC concentration of the extract and α-mangostin, respectively. We observed the re-growth which reached to the number of viable cells of 1.4 × 10^5^ and 2.2 × 10^5^ cells/mL in the presence of the extract and α-mangostin at 72 h of incubation. However, the treated cysts showed a decrease in viable cells when compared with the control that reached up to 5.8 × 10^5^ cells/mL at 72 h of incubation (Fig. 3c,d).

### Synergistic effects

**Figure 4:**
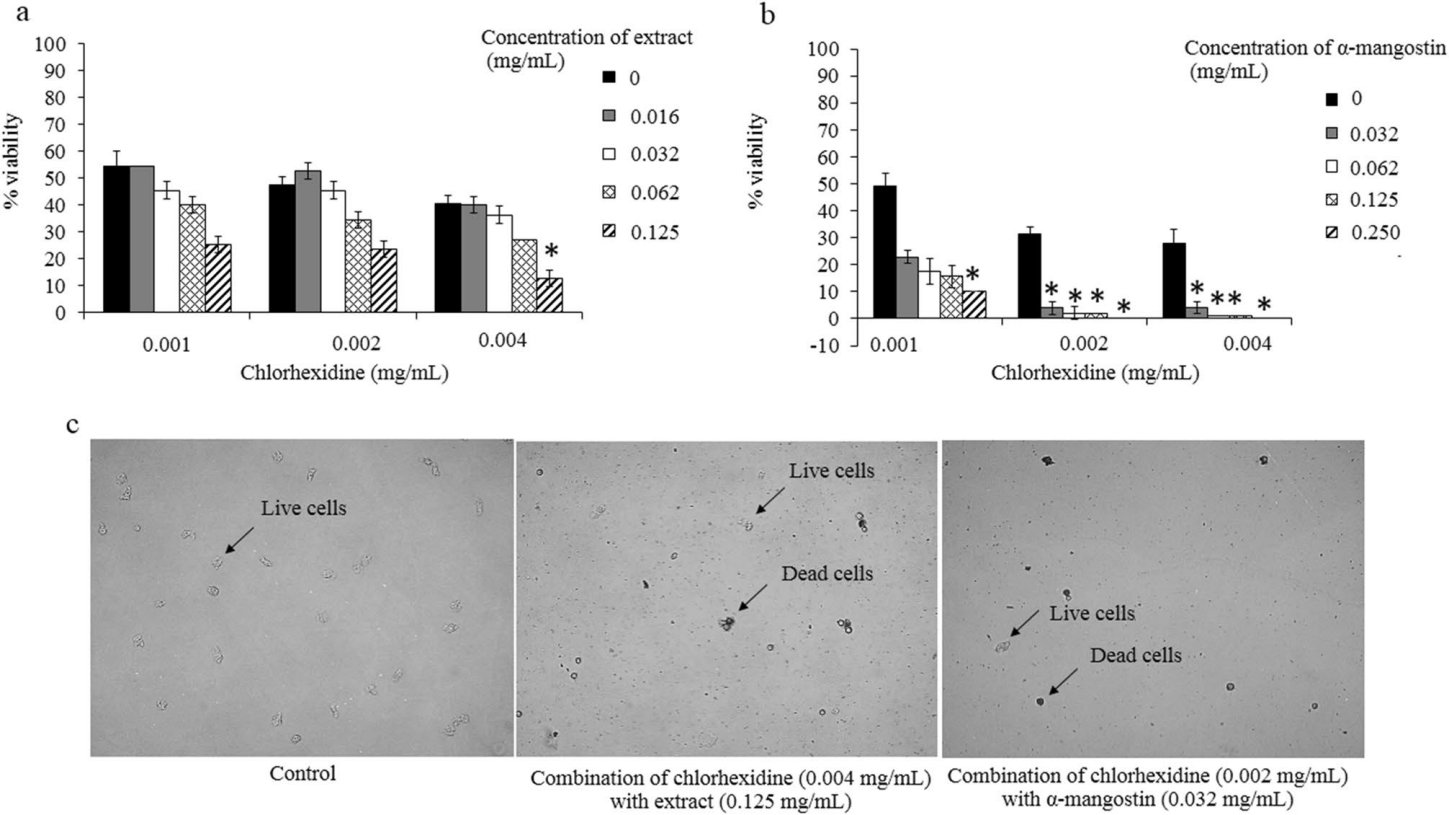
Combination of *G. mangostana* extract or α-mangostin with chlorhexidine for anti-amoebic effects on trophozoites of *A. triangularis.* Parasites were grown in PYG medium in the presence of chlorhexidine alone and combination with *G. mangostana* extract (**a**) or α-mangostin (**b**) for 24 h. The inhibitory activity was carried out using trypan blue exclusion assay; 1% DMSO was used as negative control (**c**). Treated trophozoites were observed under inverted microscopy (20x). The relative percentage of viability was defined as (mean of the treated /mean of the control) × 100. *p < 0.05, statistically significant difference in combination to single drug treatment.

**Figure 5:**
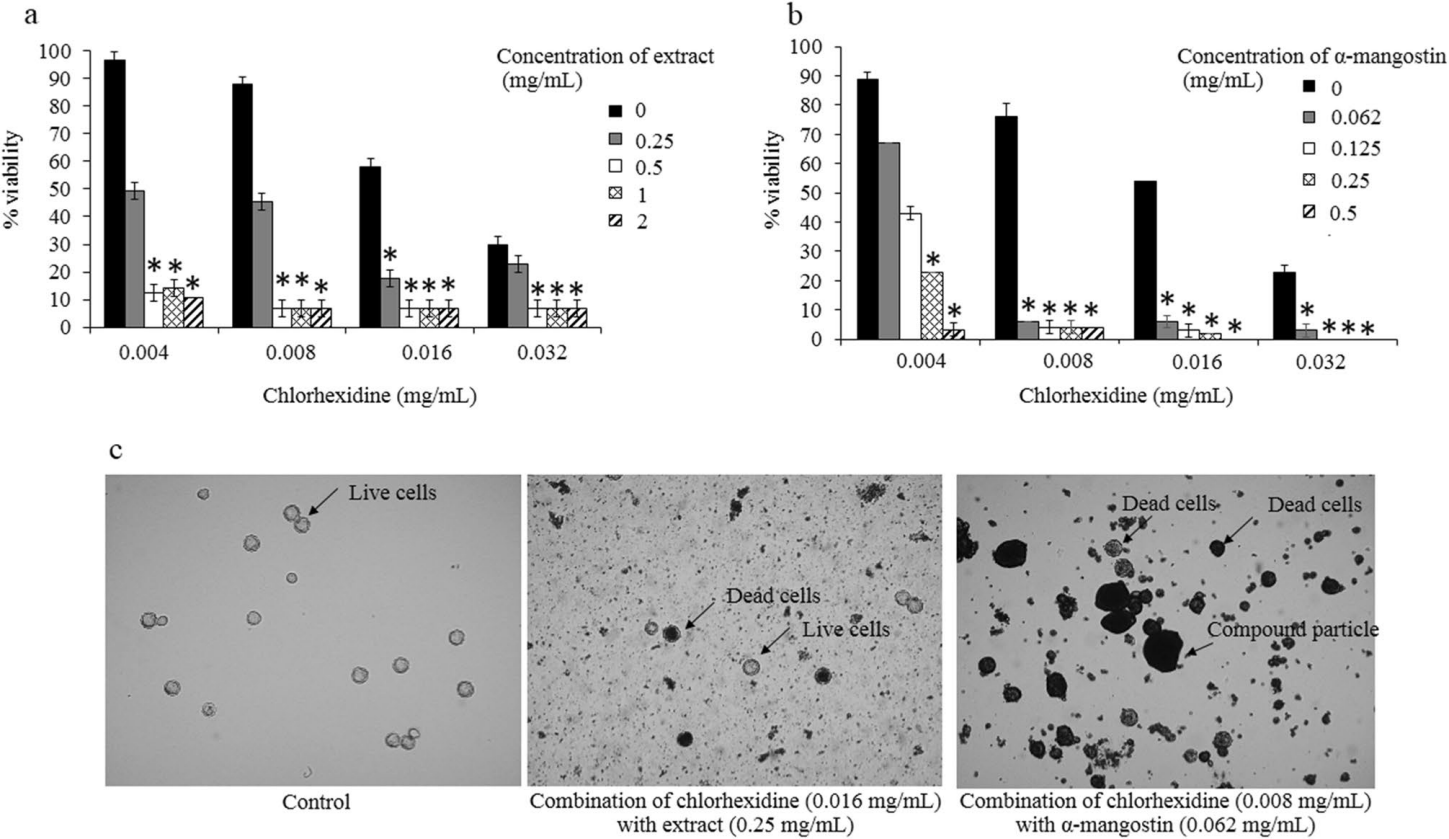
Combination of *G. mangostana* extract or α-mangostin with chlorhexidine for anti-amoebic effects on cysts of *A. triangularis.* Parasites were grown in PYG medium in the presence of chlorhexidine alone and combination with *G. mangostana* extract (**a**) or α-mangostin (**b**) for 24 h. The inhibitory activity was carried out using trypan blue exclusion assay; 1% DMSO was used as a negative control (**c**). Treated cysts were observed under inverted microscopy (40x). The relative percentage of viability was defined as (mean of the treated /mean of the control) × 100. *p < 0.05, statistically significant difference in combination to single drug treatment.

The evaluation of the synergistic effects between the plant extract and the drug was determined in this study. For *A. triangularis* trophozoites, an additive interaction (FICI = 1) was observed in the combination of chlorhexidine and the extract. Chlorhexidine at 0.002 mg/mL was synergistic when combined with α-mangostin at 0.032, 0.062 and 0.125 mg/mL (Table 2). For the cystic form, chlorhexidine was synergistic when combined with the extract and α-mangostin. With the extract, synergism was observed in 0.004, 0.008 and 0.016 mg/mL of chlorhexidine when combined with the extract 0.5 and 1 mg/mL. In addition, chlorhexidine at 0.004 and 0.008 mg/mL was found to be synergistic with various concentrations (0.062, 0.125 and 0.25 mg/mL) of α-mangostin (Table 3). The percentage of viability in *A. triangularis* in additive and synergistic effects was less than 10% (Figs. 4 and 5).

**Table 2:** Fractional inhibitory concentration index (FICI) of the combination test in targeting *A. triangularis* trophozoites.

Combination agents	MIC in combination (mg/mL)		FICI	Effect
	Chlorhexidine	Extract or α-mangostin		
*G. mangostana* extract	0.004	0.125	1	Additivity
α-mangostin	0.002	0.032	0.31	Synergistic
	0.002	0.062	0.37	Synergistic
	0.002	0.125	0.50	Synergistic

**Table 3:** Fractional inhibitory concentration index (FICI) of the combination test in targeting *A. triangularis* cysts.

Combination agents	MIC in combination (mg/mL)		FICIFICI	Effect
	Chlorhexidine	Extract or α-mangostin		
*G. mangostana* extract	0.004	0.5	0.18	Synergistic
	0.004	1	0.31	Synergistic
	0.008	0.5	0.24	Synergistic
	0.008	1	0.37	Synergistic
	0.016	0.5	0.36	Synergistic
α-mangostin	0.004	0.062	0.12	Synergistic
	0.004	0.125	0.18	Synergistic
	0.004	0.250	0.31	Synergistic
	0.008	0.062	0.18	Synergistic
	0.008	0.125	0.24	Synergistic
	0.008	0.250	0.37	Synergistic
	0.008	0.125	0.24	Synergistic
	0.008	0.062	0.18	Synergistic

### SEM analysis

**Figure 6:**
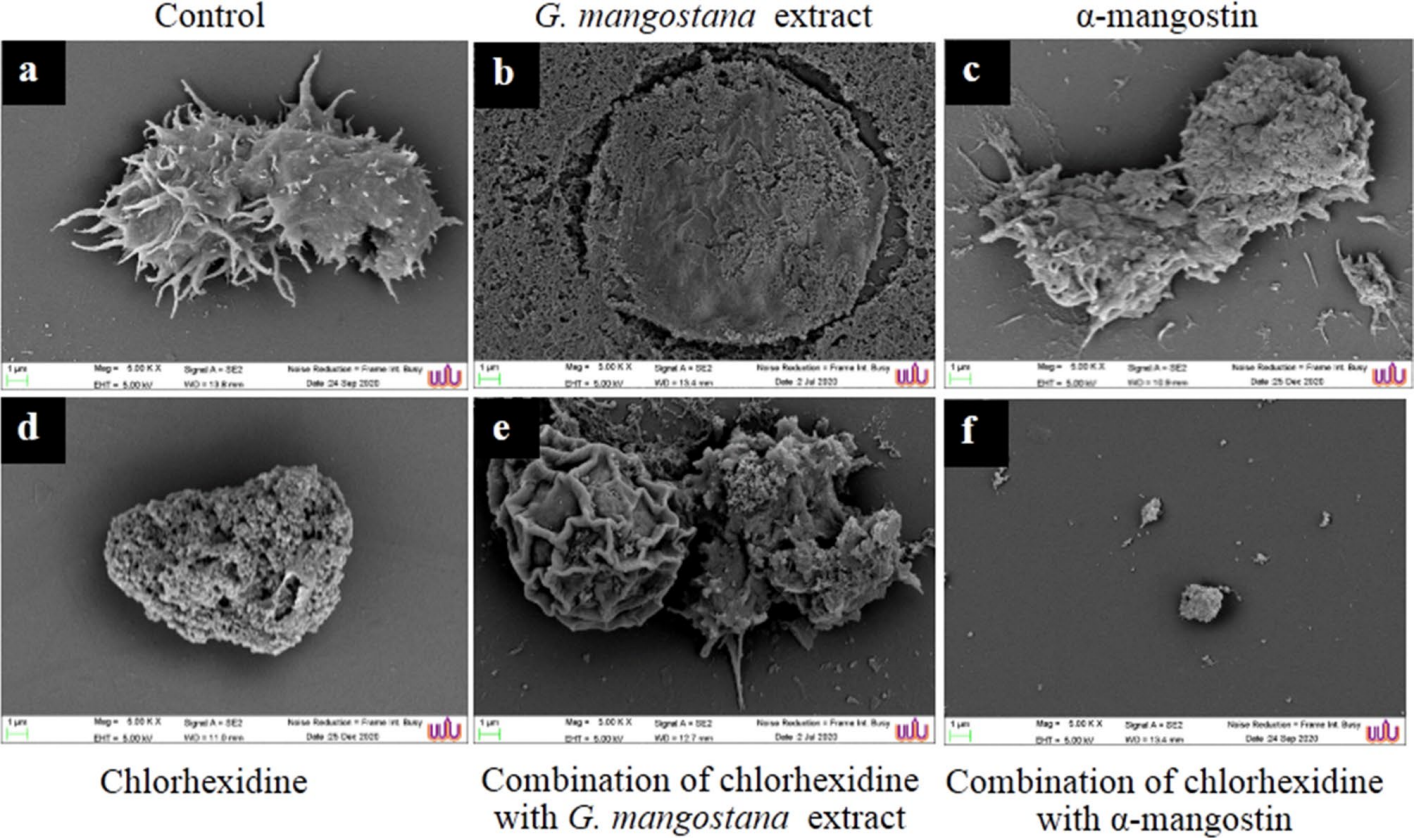
Scanning micrographs of *A. triangularis* trophozoites after treatment with *G. mangostana* extract, chlorhexidine, α-mangostin and in combination at 24 h. Control trophozoites (**a**), trophozoites treated with G*. mangostana* extract (1 mg/mL) (**b**), α-mangostin (0.5 mg/mL) (**c**), chlorhexidine (0.008 mg/mL) (**d**), and combination of chlorhexidine (0.004 mg/mL) and *G. mangostana* extract (0.125 mg/mL) (**e**), combination of chlorhexidine (0.002 mg/mL) and α-mangostin (0.032 mg/mL) (**f**). Magnification: (**a**–**f**) = × 5000.

**Figure 7:**
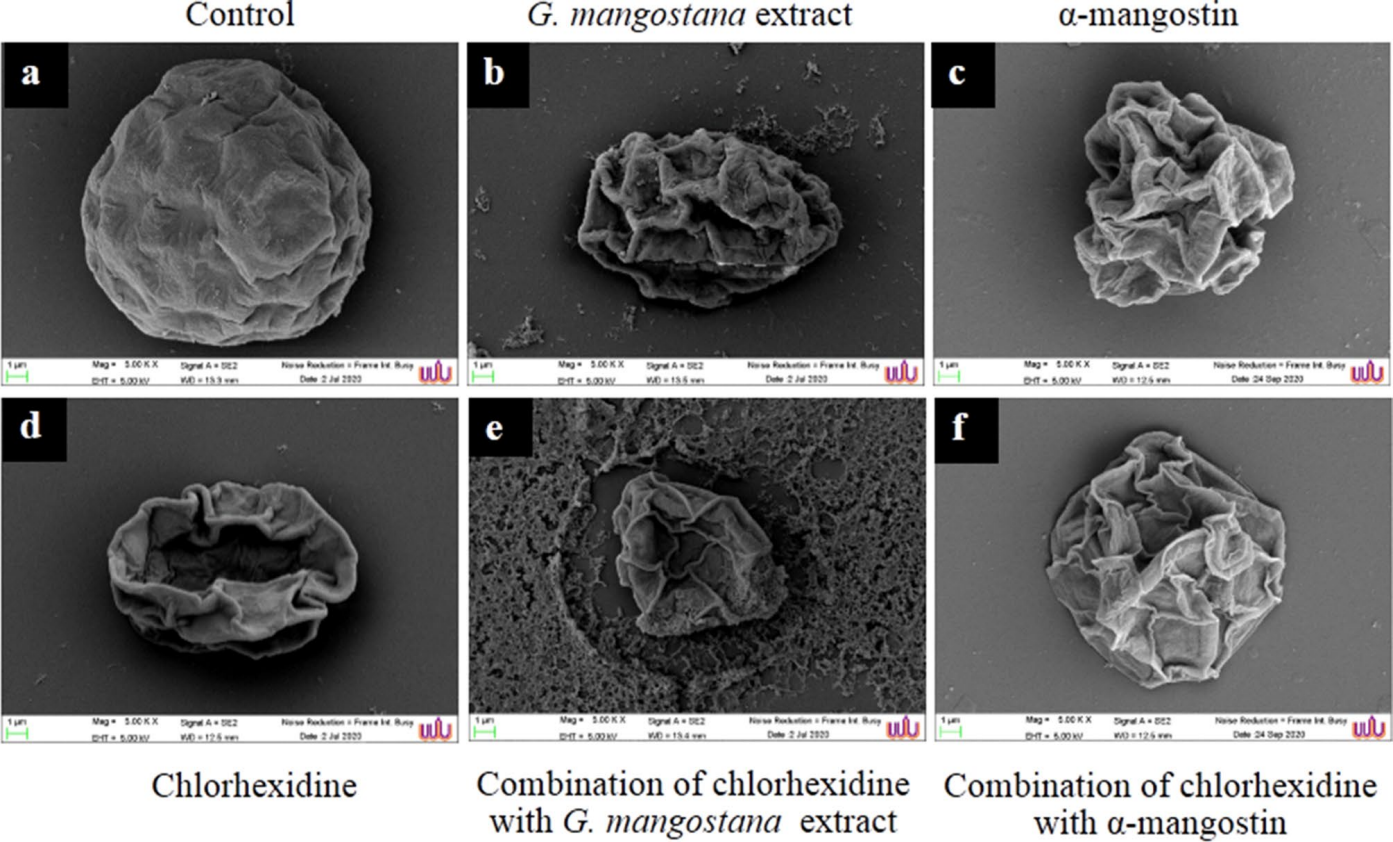
Scanning micrographs of *A. triangularis* cysts after treatment with *G. mangostana* extract, chlorhexidine, α-mangostin and in combination at 24 h. Control cysts (**a**), cysts were treated with G*. mangostana* extract (16 mg/mL) (**b**), α-mangostin (4 mg/mL) (**c**), chlorhexidine (2.56 mg/mL) (**d**), and combination of chlorhexidine (0.004 mg/mL) and *G. mangostana* extract (0.5 mg/mL) (**e**), combination of chlorhexidine (0.04 mg/mL) and α-mangostin (0.062 mg/mL) (**f**). Magnification: (**a**–**f**) = × 5000.

The alterations due to the action of *G. mangostana* extract and α-mangostin were confirmed by SEM micrograph are shown in Figs. 6 and 7. Control trophozoites (Fig. 6a) treated with 1% DMSO exhibited normal cells with acanthopodia. During the treatment with the extract (Fig. 6b), the damaged cells were observed as flat and smooth surface. For treatment with MIC of chlorhexidine and α-mangostin, the trophozoites reduced in size, shrunken appearance, loss of acanthapodia, and having pores-like structure on the surface as shown in Fig. 6c,d. Trophozoites treated with the combination of chlorhexidine and the extract at FICI equal 1, SEM showed rounded morphology with pores on the cell surface (Fig. 6e). In combination with chlorhexidine and α-mangostin, trophozoites have died completely with a rough surface and tiny when compared to the control (Fig. 6f). Also, micrographs of control cysts were intact with oval shape and smooth surface as shown in Fig. 7a. The oval cysts of *A. triangularis* were flat, irregular in shape, causing a collapse of the ectocyst walls after treatment with chlorhexidine, the extract, α-mangostin and in combination (Fig. 7b–f).

### Toxicity

At low concentrations, ranging from 0.032–0.062 mg/mL for *G. mangostana* extract, the number of living cells were constant at 24 h. However, the survival of Vero cells tended to be lower than those after treatments with the extract (0.125–4 mg/mL) (Fig. S2a). For α-mangostin, the surviving Vero cells were observed at 0.008–0.016 mg/mL (non-toxic) but decreased at the concentrations of 0.032–1 mg/mL (Fig. S2b). The effect of α-mangostin on Vero cells was further determined according to IC_50_ value, which was obtained from the viable interpolation of the cell line. Pure α-mangostin exhibited an inhibitory effect on Vero cells with IC_50_ value 0.016 mg/mL (39 μM) after 24 h of treatment (Fig. S2c). To reduce the toxicity, a combination sets of α-mangostin and chlorhexidine treatment was further challenged to determine the survival of Vero cells. However, it was clearly observed the decreased survial when the concentration of α-mangostin was higher than 0.016 mg/mL (data were not shown).

## Discussion

The herb-drug interaction may impact to the potential health-promoting effect of increasing drug efficacy or decreasing common adverse effect. As such, we present in this study an effective treatment for *A. triangularis* infection with herb-drug based combination strategy. Also, we highlight the potential of the anti-*Acanthamoeba* activity of *G. mangostana* which also known as the “queen of fruits”.

In previous literature, xanthones is a major phytochemical compound from *G. mangostana* which have been presented in several parts of plant such as pericarp, whole fruits, leaves and bark^6^. The pericarp of mangosteen fruit are the most abundant of xanthones α-mangostin (78% total xanthone content)^23^. In addition, there are various components of other xanthones, such as β-mangostin, gartanin, 8-deoxygartanin, garcinones A, B, C, D and E, mangostinone, 9-hydroxycalabaxanthone, and isomangostin^6,24^. Nevertheless, previous studies have reported that the concentration of α-mangostin correlates with the biological activity of mangosteen extract^10^. Interestingly, the compound α-mangostin from *G. mangostana* has ever been reported its property against other parasites like *Plasmodium falciparum*^22^.

Some studies have reported the effects of *G. mangostana* extract and α-mangostin on anti-parasitic activity^22,25^, but the effect on *Acanthamoeba* spp. has so far never been explored. Our study therefore demonstrated for the first time of *G. mangostana* extract from the pericarp and α-mangostin exhibit anti-*Acanthamoeba* activity against *A. triangularis* trophozoites and cysts. *A. triangularis* WU19001 was a selected strain parasite used in this study due to its characteristic property belong to group II (genotype T4). Group 2 *Acanthamoeba* species are pathogenic in nature, typically having double-walled with a wrinkled ectocyst and stellate polygonal, triangular, or oval endocyst^26^. *A. triangularis* was originally isolated from human faeces^27^ and currently discovered from environmental water souces, as reported in this study. In 2008, the pathogenic strain of *A. triangularis* has firstly reported in a clinically confirmed case of *Acanthamoeba* keratitis in Korea^28^. Due to climate change and global warming, it is postulated that *A. triangularis* would be more likely to be associated to clinical scenario in times to come as it is widely contaminated in the environment.

In fact, eradicating *Acanthamoeba* infection seems impossible due to the high resistance of the cysts to anti-*Acanthamoeba* drugs. Therefore, we also investigated the effective concentration of *G. mangostana* extract and α-mangostin against *A. triangualris* trophozoite and cyst stages. Our results showed that the extract and α-mangostin can inhibit *A. triangularis* in PYG medium. It is noteworthy that the number of *Acanthamoeba* trophozoites and cysts were significantly reduced (*p* < 0.05) in the treatment with MIC concentrations of the extract and α-mangostin. They were found to have the greatest growth-inhibitor for trophozoites at 72 h. For the cystic form, they survived on 72 h (Fig. 3) which might be due to the decreased the decreased effect of the extract and α-mangostin. In addition, the cyst form is a is a dormant stage against severe condition, including the presence of anti-*Acanthamoeba* agents^29^. Overall, this study therefore suggests that the extract and α-mangostin are promising agents that shows the remarkable effects against *A. triangularis* infections. However, the development method for support the stability of the extract and α-mangostin has been validated since it is important for further pharmacokinetic or tissue distribution ex vivo studies.

Chlorhexidine is the drug of choice for treatment *Acanthamoeba* keratitis, since it is effective against both trophozoites and cysts^8^. In this study, a single drug chlorhexidine was studied on anti-*Acanthamoeba* activity and prosperously exhibited inhibitory activity in *A. triangularis* trophozoites and cysts with MIC values of 0.008 and 0.064 mg/mL (Table 1), respectively. According to an earlier report, chlorhexidine showed amoebicidal and cysticidal properties at 200 μg/mL (0.02%), but it exhibited side effects^30^. In fact, a single drug, used to treat infectious diseases, includes this parasitic infection that causes side effects, long-term for clearance use, high cost and drug-resistant parasites. Therefore, the combination approach is constantly being introduced to find these pitfalls. Chlorhexidine has often been used in combination with aromatic diamidines^8^, aminoglycosides, imidazoles, and polyene^31^. However, these chemicals have side effects on keratocytes found in cases of human keratitis^32^. Recently, the discovery of compounds with anti-*Acanthamoeba* activity in plants and herbs has been very encouraging to evaluate a source of secondary metabolites with anti-*Acanthamoeba* effects. The combination test for possible synergistic effects against *Acanthamoeba* spp. was considered to reduce the MIC of the drug. To support this, our study also revealed the effect of the combination of *G. mangostana* extract and chlorhexidine against *A. triangularis* trophozoites and cysts. Chlorhexidine showed an additive effect when combined with the *G. mangostana* extract. An additive effect occurs when the substance added to increase or improve the effectiveness but not to the extent of synergistic interaction^33^. *G. mangostana* extract was combined with chlorhexidine to produce a synergistic effect against *A. triangularis* cysts. The FICI values demonstrated the synergy for concentration of 0.004 to 0.016 mg/mL of chlorhexidine and 0.5 to 1 mg/mL of *G. mangostana* extract as shown the viability of less than 10%. The α-mangostin was found to be more effective when combined with chlorhexidine. It is interesting to note that the concentration of chlorhexidine can be reduce by 1/4–1/16 of the MIC in the presence of α-mangostin. Nowadays, it is difficult and expensive to develop new drugs, therefore it has been considered the finding of alternative strategies to reduce toxicity and/or the development of resistance pathogens. From this study, it appears to be a promising combined chlorhexidine with *G. mangostana* extract or α-mangostin to fight infection and especially the resistance pattern of *Acanthamoeba* spp. in the future.

The mode of action considered in this study was confirmed by scanning electron microscopy (SEM), as shown in Figs. 6 and 7. Treated trophozoites showed similar flat cells and smooth surfaces as a result of the total destruction of acanthopodia in the presence of extract and chlorhexidine. Regarding the combination of the drug and *G. mangostana* extract or α-mangostin, the morphology of trophozoites being observed in the presence of pores on their surface and cells were rounded and small. Chlorhexidine is positively and ionically charged with the parasite´s negatively charged plasma membrane, resulting in the membrane structure that gives rise to permeability modulation, ionic leakage and cytoplasmic disruptions causing cellular damage and cell death^34,35^**.** Control cysts showed regular morphological characteristics. Overall, *A. triangularis* cysts were flat and morphologically deformed (of irregular shape and size) as a result of the destruction of the ectocyst walls after the treatment given with *G. mangostana* extract, α-mangostin, chlorhexidine and in combination.

For a pure compound, α-mangostin exhibited toxicity to Vero cells at IC_50_ values 39 μM (0.016 mg/mL). Our preliminary finding is however surprisingly different from a previous study reported that α-mangostin was non-toxic to nontumorigenic human pancreatic duct epithelial cells, at a dose as high as 40 μM^36^. In addition, xanthones in *G. mangostana* pericarp proved to be non-toxic to mice in vivo when administered orally at a dose of 100 mg/kg of body weight/day for 7 days^37^. Since α-mangostin is gaining more popular to be used against infectious diseases, therefore, it strongly suggests for further comprehensive studies such as chemical structure modification and structure–activity relationship as well as nanotechnology to evaluate the mechanism of action of these compounds against *Acanthamoeba* spp., before any conclusion could be made.

Besed on the results obtained from this study, ethanolic extract of *G. mangostana* from the pericarp and α-mangostin possess anti-*Acanthamoeba* activities against *A. triangularis* trophozoite and cyst stages. Moreover, the present study focuses on the novel combination for treatment of *Acanthamoeba* infection. The combination of *G. mangostana* extract and α-mangostin with chlorhexidine generated synergistic effects which increased effectively for the treatment of *Acantrhamoeba* infection.

## Materials and methods

### Preparation of plant extracts

The 50 g of dry *G. mangostana* pericarp powder was soaked in 200 mL of ethanol for 7 days. The extract was filtered through Whatman No. 1 (GE Healthcare Life Science, Buckinghamshire HP7 9NA, United Kingdom) using a vacuum and pressure pump. The solution was evaporated to dryness under reduced pressure using a rotary evaporator to obtain *G. mangostana* extract. The 200 mg of extract was subjected to column chromatography using silica gel as a stationary phase and eluted with 20% acetone in 750 mL of hexane. The collected fractions were chromatographed on a silica gel thin-layer chromatography compared with the authentic compound, α-mangostin. Fractions with one spot of α-mangostin on chromatogram were combined. The solvent was removed to give 30 mg yellow solid of α-mangostin. The extract and α-mangostin were dissolved in 99.5% DMSO and stored at − 20 °C until use.

### Cultivation of *A. triangularis*

*A. triangularis* WU19001, a strain from the recreational reservoir at Walailak University, Nakhon Si Thammarat-Thailand, was used in this study^38^. The parasite was grown in PYG medium [20 g proteose peptone, 2 g yeast extract, 0.98 g MgSO_4_·7H_2_O, 0.35 g Na_2_HPO_4_·7H_2_O, 0.34 g KH_2_PO_4_, 0.02 g (NH_4_)_2_Fe(SO_4_)_2_·6H_2_O, 18 g glucose]. The trophozoites were observed after 72 h of incubation at room temperature and were cultured in this medium for 1 week The 90% mature cysts were obtained PYG medium. The cysts were harvested when the cultures were incubated for at least 1 week without addition fresh medium. The parasite reproduced exponentially until they reached the maximum level of 1 × 10^6^ cells/mL, after which a reduced nutrients led to encystation (cyst formation) due to unfavorable conditions for the parasite’s growth especially into trophozoite stage. At the end, all fully homogenic inoculum of mature cysts were successfully harvested. Trophozoites and cysts were centrifuged at 4000 rpm for 5 min and re-suspended in fresh PYG thereafter. For counting, 50 μL of cell suspension was mixed with 50 μL of trypan blue. Viability was investigated under the inverted microscope based on the principle of the dye can cross the membrane of dead cells with blue color, but not intact membrane of viable cells with colorless appearances.

### Minimal inhibitory concentration (MIC)

The minimum inhibitory concentration (MIC) for *G. mangostana* extract and α-mangostin was determined using the microtiter broth dilution method^38^. The extract and α-mangostin were diluted to give a final concentration of 4, 2, 1, 0.5, 0.25, 0.125, 0.062 mg/mL in a 96-well microplate. Then 100 µL of 2 × 10^5^ cells/mL of trophozoites and cysts were inoculated into each well. Chlorhexidine and 1% DMSO were included as a positive and negative control, respectively. The plates were incubated at room temperature for 24 h. The viability of parasites was calculated as follows: % viability = (mean of the viable parasite/control) × 100. The MIC value was defined as the lowest concentration that inhibited > 90% of viable growth when compared with the control.

### Growth assay

In the present study, the growth inhibition on *A. triangularis* of *G. mangostana* extract and α-mangostin was carried out following the procedure that previously described^1^ with modifications. The trophozoites and cysts (2 × 10^5^ cells/mL) were incubated with the extract and α-mangostin in the MIC, 0.5 × MIC and 0.25 × MIC, except for untreated control tubes, which had only PYG medium and incubated at room temperature for 72 h. At 24 h intervals, the viability of parasite was determined by staining with 0.2% trypan blue.

### Drug combinations

The checkerboard method^39^ was used to evaluate the interaction between *G. mangostana* extract/α-mangostin and chlorhexidine against *A. triangularis*. Subsequently, the microdilution assay was performed in a 96-well plate with a final volume of 200 µL. The extract, α-mangostin and chlorhexidine were diluted with PYG to obtain 4 times to their final concentrations of 1/16 MIC, 1/8 MIC, 1/4 MIC, 1/2 MIC and MIC. A total of 100 µL of the extract + chlorhexidine or α-mangostin + chlorhexidine were prepared in 96-well plate and added 100 µL of parasite suspension containing 2 × 10^5^ cells/mL into the wells. The plates were incubated at room temperature for 24 h. The viability of parasite was defined as the lowest concentration that inhibited > 90% of growth when compared to the negative control. The assessment of the results was defined as the Fractional Inhibitory Concentration Index (FICI), which was calculated using the following:

$${\text{FICI}}\;{\text{of}}\;{\text{combination}} = {\text{FIC}}\;{\text{A}} + {\text{FIC}}\;{\text{B}}$$

$${\text{FIC}}\;{\text{A}} = {\text{MIC}}\;{\text{of}}\;{\text{chlorhexidine}}\;{\text{in}}\;{\text{combination}}/{\text{MIC}}\;{\text{of}}\;{\text{chlorhexidine}}\;{\text{alone}}$$

$${\text{FIC}}\;{\text{B}} = {\text{MIC}}\;{\text{of}}\;{\text{extract}}\;{\text{in}}\;{\text{combination}}/{\text{MIC}}\;{\text{of}}\;{\text{extract}}\;{\text{alone}}.$$

The combination was considered synergistic for FICI ≤ 0.5, additive for 0.5 < FICI ≤ 1, indifferent for 1 < FICI < 4, and antagonistic for FICI ≥ 4, according to European Committee on Antimicrobial Susceptibility Testing (EUCAST) definition.

### Scanning electron microscopic (SEM) study

Trophozoites and cysts of *A. triangularis* were treated with chlorhexidine, *G. mangostana* extract, α-mangostin and in combinations. After incubation, cells were collected by centrifugation at 4000 rpm for 5 min and re-suspended in phosphate buffer saline (PBS). Cells in 1% DMSO were used as negative controls. Samples were fixed with 2.5% glutaraldehyde overnight. The samples were further dehydrated with a series of graded alcohol (20%, 40%, 60%, 80%, 90%, and 100% ethanol), mounted on aluminum stubs, and allowed to dry using a critical point dryer. Samples were then coated with gold particles and the morphology of *A. triangularis* trophozoites and cysts after treatment was subsequently examined under SEM (SEM-Zeiss, Munich, Germany) at the Center for Scientific and Technological Equipment, Walailak University, Nakhon Si Thammarat, Thailand^38^.

### Toxicity

The cytotoxic effects of *G. mangostana* extract, α-mangostin, chlorhexidine and combination sets were evaluated using the Vero cell line (8200F270602). Cells were cultured in Dulbecco's Modified Eagle's (DMEM) medium (Merck KGaA, Darmstadt, Germany) supplemented with 10% FBS and 1% antibiotic containing penicillin G of 100 units/mL, streptomycin of 100 μg/mL. The culture was incubated at 37 °C in a humidified atmosphere and 5% CO_2_. After the cells reached 90% confluence, detachment was performed using trypsin ethylene diamine tetraacetic acid (EDTA), incubated at 37 °C in 5% CO_2_. Single cells at a density of 1.5 × 10^4^ cells/100 μL were seeded into each well of a 96-well polystyrene plate and allowed to attach for 24 h. Then, 100 μL of the extract and α-mangostin, previously prepared at multiple concentrations, chlorhexidine and combination sets were gently added. After incubation for 24 h, the cytotoxic effects were determined using the MTT assay. The absorbance was measured using a microplate reader (Biotek, Cork, Ireland) at 570 nm. The percent survival was calculated using the following equation:

$$\% \;{\text{survival}} = \left( {{\text{ABt}}/{\text{ABu}}} \right) \times {1}00$$

ABt and ABu denote the absorbance values of treated and untreated cells, respectively^40^.

### Statistical analysis

The experiments were performed in triplicate. All data were recorded and entered using the statistical package software (SPSS Inc. Chicago, IL, USA). The data were expressed as mean ± SD. Statistical analysis was analyzed by the two-tailed unpaired Student’s t-test. In all analyzes, *p* < 0.05 was considered statistically significant.

## Supplementary Information

Supplementary Information.

## References

[1] Chu D, Miles H, Toney D, Ngyuen C, Marciano-Cabral F (1998). Amebicidal activity of plant extracts from Southeast Asia on *Acanthamoeba* spp. Parasitol. Res..

[2] Sifaoui I (2017). Evaluation of the anti-*Acanthamoeba* activity of two commercial eye drops commonly used to lower eye pressure. Exp. Parasitol..

[3] Tice AK (2016). Expansion of the molecular and morphological diversity of Acanthamoebidae (Centramoebida, Amoebozoa) and identification of a novel life cycle type within the group. Biol. Direct..

[4] Haniloo A, Pezeshki A, Mahmmodzadeh A, Kadkhodamohammadi E (2017). Genotyping of *Acanthamoeba* spp. from water sources from Northwestern Iran. Acta Parasitol..

[5] Coronado-Velázquez D (2019). *Acanthamoeba mauritaniensis* genotype T4D: An environmental isolate displays pathogenic behavior. Parasitol. Int..

[6] Chao M, Thongseesuksai T, Boonmars T, Laummaunwai P (2020). Investigation of the *in vitro* cysticidal activity of miltefosine against *Acanthamoeba* spp. J. Parasites Dis..

[7] Walochnik J (2008). Granulomatous amoebic encephalitis caused by *Acanthamoeba* amoebae of genotype T2 in a human immunodeficiency virus-negative patient. J. Clin. Microbiol..

[8] Lorenzo-Morales J, Khan NA, Walochnik J (2015). An update on *Acanthamoeba*keratitis: Diagnosis, pathogenesis and treatment. Parasites.

[9] Taher M, Tg Zakaria T, Susanti D, Zakaria ZA (2016). Hypoglycaemic activity of ethanolic extract of *Garcinia mangostana* Linn. in normoglycaemic and streptozotocin-induced diabetic rats. BMC Complement. Altern. Med..

[10] Ibrahim MY, Hashim NM, Mariod AAA (2016). α-mangostin from *Garcinia mangostana* Linn: An updated review of its pharmacological properties. Arab. J. Chem..

[11] Sunarjo L, Suharti O, Susanto HS (2017). The preliminary study on safety of using mangosteen peel extract as natural herbs. JMSCR.

[12] Sakagami Y, Iinuma M, Piyasena KG, Dharmaratne HR (2005). Antibacterial activity of alpha-mangostin against vancomycin resistant Enterococci (VRE) and synergism with antibiotics. Phytomedicine.

[13] Puripattanavong JKW, Khajorndetkun W, Chansathirapanich W (2006). Improved isolation of α-mangostin from the fruit hull of *Garcinia mangostana*and its antioxidant and antifungal activity. Planta Med..

[14] Husen SA, Khaleyla F, Ansori ANM, Susilo RJK, Winarni D (2018). Antioxidant activity assay of alpha-mangostin for amelioration of kidney structure and function in diabetic mice. ASSEHR.

[15] Aisha AF, Abu-Salah KM, Ismail Z, Majid AM (2012). *In vitro* and *in vivo* anti-colon cancer effects of *Garcinia mangostana* xanthones extract. BMC Complement. Altern. Med..

[16] Wang JJ, Sanderson BJ, Zhang W (2011). Cytotoxic effect of xanthones from pericarp of the tropical fruit mangosteen (*Garcinia mangostana* Linn.) on human melanoma cells. Food Chem. Toxicol..

[17] Matsumoto K (2003). Induction of apoptosis by xanthones from mangosteen in human leukemia cell lines. J. Nat. Prod..

[18] Chen LG, Yang LL, Wang CC (2008). Anti-inflammatory activity of mangostins from *Garcinia mangostana*. Food Chem. Toxicol..

[19] Fu Y, Zhou H, Wang M, Cen J, Wei Q (2014). Immune regulation and anti-inflammatory effects of isogarcinol extracted from *Garcinia mangostana* L. against collagen-induced arthritis. J. Agric. Food Chem..

[20] Riscoe M, Kelly JX, Winter R (2005). Xanthones as antimalarial agents: Discovery, mode of action, and optimization. Curr. Med. Chem..

[21] Azebaze AG, Meyer M, Valentin A, Nguemfo EL, Fomum ZT, Nkengfack AE (2006). Prenylated xanthone derivatives with antiplasmodial activity from *Allanblackia monticola* STANER L.C. Chem. Pharm. Bull..

[22] Mahabusarakam W, Kuaha K, Wilairat P, Taylor WC (2006). Prenylated xanthones as potential antiplasmodial substances. Planta Med..

[23] Ansori ANM (2020). A review on medicinal properties of mangosteen (*Garcinia mangostana* L.). Res. J. Pharm. Technol..

[24] Gutierrez-Orozco F, Failla ML (2013). Biological activities and bioavailability of mangosteen xanthones: A critical review of the current evidence. Nutrients.

[25] Upegui Y (2015). *In vivo* antimalarial activity of α-mangostin and the new xanthone δ-mangostin. Phytother Res..

[26] Marciano-Cabral F, Cabral G (2003). *Acanthamoeba* spp. as agents of disease in humans. Clin. Microbiol. Rev..

[27] Pussard M, Pons R (1977). Morphologie de la paroi kystique et taxonomie du genre *Acanthamoeba* (Protozoa, Amoebida). Protistologica.

[28] Xuan YH, Chung BS, Hong YC, Kong HH, Hahn TW, Chung DI (2008). Keratitis by *Acanthamoeba triangularis*: Report of cases and characterization of isolates. Korean J. Parasitol..

[29] Bunsuwansakul C (2019). *Acanthamoeba* in Southeast Asia: Overview and Challenges. Korean J. Parasitol..

[30] Siddiqui R, Aqeel Y, Khan NA (2016). The development of drugs against *Acanthamoeba* infections. Antimicrob. Agents Chemother..

[31] Fakae LB, Stevenson CW, Zhu XQ, Elsheikha HM (2020). *In vitro* activity of *Camellia sinensis* (green tea) against trophozoites and cysts of *Acanthamoeba castellanii*. Int. J. Parasitol. Drugs Drug Resist..

[32] Anwar A (2020). Antiamoebic activity of synthetic tetrazoles against *Acanthamoeba castellanii* belonging to T4 genotype and effects of conjugation with silver nanoparticles. Parasitol. Res..

[33] Cheesman MJ, Ilanko A, Blonk B, Cock IE (2017). Developing new antimicrobial therapies: Are synergistic combinations of plant extracts/compounds with conventional antibiotics the solution?. Pharmacogn. Rev..

[34] Elsheikha HM, Siddiqui R, Khan NA (2020). Drug discovery against *Acanthamoeba* infections: Present knowledge and unmet needs. Pathogens.

[35] Fatimah H, Nakisah MA (2013). Visualization on the effect of chlorhexidine gluconate, a biocide on *Acanthamoeba* sp. by electron microscopy. Malays. J. Microsc..

[36] Hafeez BB (2014). α-Mangostin: a dietary antioxidant derived from the pericarp of *Garcinia mangostana* L. inhibits pancreatic tumor growth in xenograft mouse model. Antioxid. Redox Signal..

[37] Kaomongkolgit R, Jamdee K, Pumklin J, Pavasant P (2013). Laboratory evaluation of the antibacterial and cytotoxic effect of alpha-mangostin when used as a root canal irrigant. Indian J Dent..

[38] Mitsuwan W (2020). *Curcuma longa* ethanol extract and *Curcumin* inhibit the growth of *Acanthamoeba triangularis* trophozoites and cysts isolated from water reservoirs at Walailak University,Thailand. Pathog Glob. Health..

[39] Lorian V, Lorian V (1996). Antibiotics. Laboratory Medicine.

[40] Kaomongkolgit R, Jamdee K, Chaisomboon N (2009). Antifungal activity of alpha-mangostin against *Candida albicans*. J. Oral Sci..

