# Supplementary material for: Anti-Acanthamoeba synergistic effect of chlorhexidine and Garcinia mangostana extract or α-mangostin against Acanthamoeba triangularis trophozoite and cyst forms
Source: Sci Rep. 2021 Apr 13;11:8053. doi: 10.1038/s41598-021-87381-x (PMC8044166; doi:10.1038/s41598-021-87381-x)
Supplement: Supplementary file 1 — Supplementary Information. [file 41598_2021_87381_MOESM1_ESM.pdf]

Anti-*Acanthamoeba* synergistic effect of chlorhexidine and *Garcinia mangostana* extract or α-mangostin against *A. triangularis* trophozoite and cyst forms

Suthinee Sangkanu[1], Watcharapong Mitsuwan[1,2], Wilawan Mahabusarakam[3], Tajudeen O. Jimoh[4,5], Polrat Wilairatana[6*], Ana Paula Girol[7], Ajoy K. Verma[8], Maria de Lourdes Pereira[9], Mohammed Rahmatullah[10], Christophe Wiart[11], Abolghasem Siyadatpanah[12], Roghayeh Norouzi[13],Veeranoot Nissapatorn[1]*

[1]School of Allied Health Sciences, Southeast Asia Water Team (SEA Water Team), World Union for Herbal Drug Discovery (WUHeDD), and Research Excellence Center for Innovation and Health Products (RECIHP), Walailak University, Nakhon Si Thammarat, Thailand.

[2]Akkhraratchakumari Veterinary College, Walailak University, Nakhon Si Thammarat, Thailand.

[3]Division of Physical Science, Faculty of Science, Prince of Songkla University, Songkhla. Thailand.

[4]Department of Pharmacognosy and Pharmaceutical Botany, Faculty of Pharmaceutical Sciences, Chulalongkorn University, Bangkok, Thailand.

[5]Department of Biochemistry, Habib Medical School, Islamic University in Uganda, Kampala, Uganda.

[6]Department of Clinical Tropical Medicine, Faculty of Tropical Medicine, Mahidol University, Bangkok, Thailand.

[7]Department of Biology, Faculty of Sciences, São Paulo State University, São Paulo, Brazil.

[8]Department of Microbiology, National Institute of Tuberculosis & Respiratory Diseases (NITRD), New Delhi, India

[9]CICECO-Aveiro Institute of Materials & Department of Medical Sciences, University of Aveiro, Aveiro, Portugal

[10]Department of Biotechnology & Genetic Engineering, University of Development Alternative Lalmatia, Dhaka, Bangladesh.

[11]School of Pharmacy, University of Nottingham Malaysia Campus, Selangor, Malaysia

[12]Ferdows School of Paramedical and Health, Birjand University of Medical Sciences, Birjand, Iran.

[13]Department of Pathobiology, Faculty of Veterinary Medicine, University of Tabriz, Tabriz, Iran

Corresponding author:

Polrat Wilairatana

Department of Clinical Tropical Medicine

Faculty of Tropical Medicine

Mahidol University, Bangkok

Thailand.

Email: polrat.wil@mahidol.ac.th

Veeranoot Nissapatorn

School of Allied Health Sciences

Walailak University

Nakhon Si Thammarat, Thailand.

E-mail: nissapat@gmail.com OR veeranoot.ni@wu.ac.th

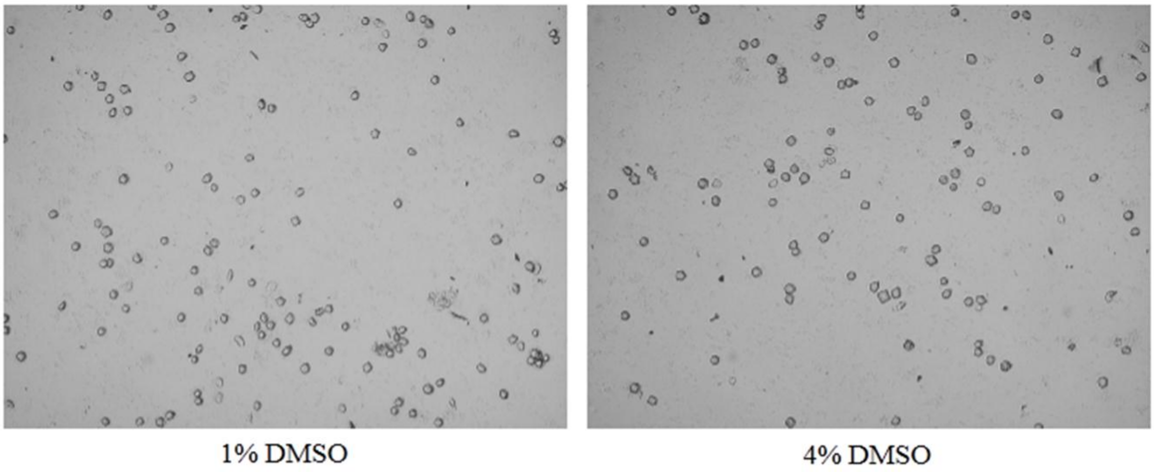

Supplementary Figure 1. Effects of DMSO on the growth and morphology of *A. triangularis* cysts. The parasites were cultured in PYG medium supplemented with 1% and 4% DMSO, incubated for 24 hours. This concentration of DMSO did not affect the growth and morphology of the cysts as observed under inverted microscope.

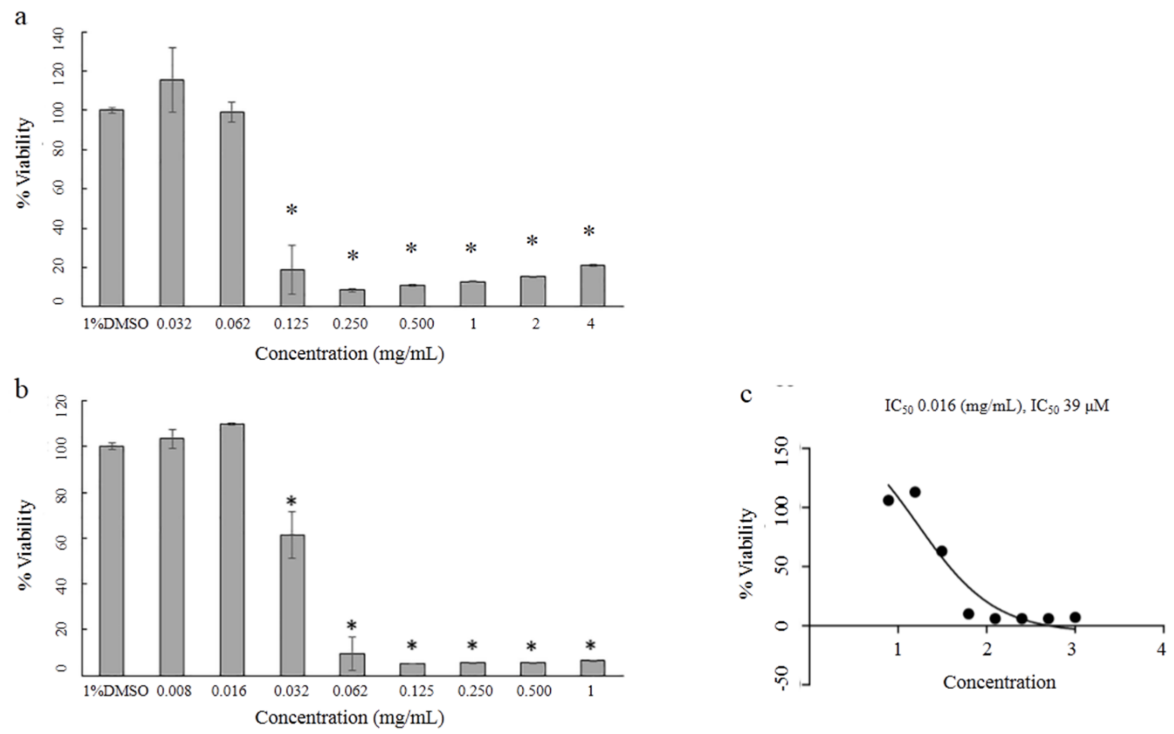

Supplementary Figure 2. Effects of *G. mangostana* extract (a) and α-mangostin (b) on viability of Vero cells using MTT assay. IC_{50} of α-mangostin (c). The cells were treated with the extract and the pure compound at different concentrations, and incubated at 37°C for 24 hours. Data are presented as a mean ± SD, (* significant difference; $p < 0.05$).

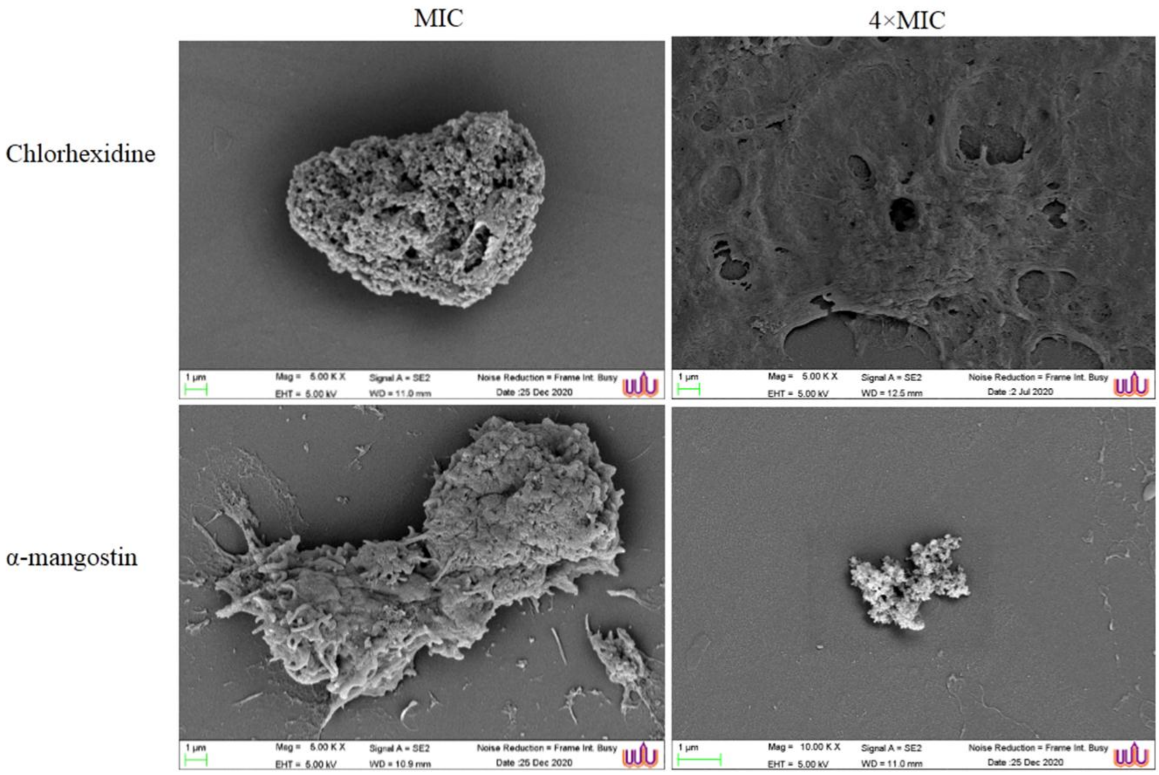

Supplementary Figure 3. Effects of the different concentrations of chlorhexidine and α-mangostin on morphology of *Acanthamoeba triangularis* WU19001 trophozoites.
